# Identifying and Evaluating Online Kidney Stone Pain Resources: A Cross-Sectional Study

**DOI:** 10.7759/cureus.73140

**Published:** 2024-11-06

**Authors:** Christopher J Hernandez, Jonathan Lim, Rebecca Takele, Frankie Escobedo, Georgina Dominique, Leslie Ojeaburu, Kymora Scotland

**Affiliations:** 1 Department of Urology, David Geffen School of Medicine, Los Angeles, USA; 2 Department of Urology, University of California Los Angeles (UCLA), Los Angeles, USA

**Keywords:** kidney stone, kidney stone pain, kidney stone resources, nephrolithiasis, stent pain, stent-related-pain

## Abstract

Introduction and objectives

With the continuous growth of social media platforms, an increasing number of individuals are turning to them as their main source of medical information. This article aims to pinpoint the most widely accessed online resources on kidney stone pain among those who suffer from kidney stones and to assess the reliability, understandability, quality, and actionability of their content.

Materials and methods

The social media analysis platform BuzzSumo was employed to identify pertinent articles and assess their engagement levels. The DISCERN instrument was utilized to evaluate the quality of the top 10 most popular articles, while online software for determining reading grade levels was used to assess article readability. Additionally, the PEMAT (Patient Education Material Assessment Tool) was employed to gauge the actionability and understandability of these articles.

Results

The BuzzSumo results were obtained through four search categories: “passing stones,” “home remedies,” “stent pain,” and “back pain.” DISCERN exhibited a mean article score of 2.67/5, indicating low quality. The average reading grade level for articles was 10.4, with a median of 10. PEMAT results indicated an average understandability score of 60%, signifying that most articles were not easily understandable, and an average actionability score of 31%, indicating a lack of actionable steps in the majority of articles to improve health outcomes.

Conclusions

Online resources about kidney stone pain were found to have shortcomings, including content with a reading level higher than average, and a lack of actionable solutions for improving overall health.

## Introduction

Patient education is foundational for optimal patient outcomes. Shared decision-making (SDM) is becoming commonplace in the medical field, and patients are increasingly expected to take an active role in participating in their treatment decisions [1]. Presently, patients can choose from a multitude of sources to learn more about their condition. Their leading options include their physician, online websites, family members, and fellow patients [2]. While physicians are an excellent source of health information, patients rarely use this resource because providers are hard to communicate with amidst their busy clinical schedules. As a result, patients often supplement medical advice from their physician with information available online. In particular, online platforms are proving to be a dominant source of medical information, with 76% of patients looking online for health-related advice [3].

Online research allows patients to access a substantial bank of information from across the world. However, this excess of information does not always result in a positive impact. Biased and misinformative online medical information is a major concern [4]. Patients may also be unable to digest or understand complex information and advice on the internet [5]. In the United States, the average reading grade level is at the eighth grade [6]. To accommodate, the National Institutes of Health (NIH) and Centers for Disease Control and Prevention (CDC) recommend that patient education materials be written at or below a sixth- to eighth-grade reading level [7]. Yet, many online sources require consumers to read at a high school grade level, rendering the wealth of online information effectively inaccessible to the majority of patients [5].

Kidney stone pain is one of the most pervasive and well-known symptoms [8] of nephrolithiasis; thus, it is the primary focus of this research project. Numerous online resources provide various medical advice to alleviate the pain of kidney stones and improve patient quality of life. As patients are increasingly relying on the internet to gather health information and aid in decision-making [3], it is crucial to assess the accuracy and comprehensibility of this content. We aim to identify and evaluate popular online kidney stone pain resources.

## Materials and methods

Google Trends

Google Trends [9] was queried to analyze the historical search data for the preliminary terms related to “kidney stone pain.” The search was limited to the time frame of 2004-2023, focused on the United States, and restricted to the English language. The program then generated a graph illustrating the overall trends for the specified phrase, based on these parameters.

BuzzSumo

Google Trends evaluation revealed the following set of keywords and phrases relating to kidney stone pain: “passing stones,” “home remedies,” “stent pain,” and “back pain.” These terms were entered into the internet analytic engine BuzzSumo, and the results were limited to written materials, excluding video content, to focus on assessing the understandability and reliability of the content. All the articles analyzed in this study were published between February 2016 and February 2023. BuzzSumo produced the corresponding engagement data for each article analyzed. Engagement levels are defined by the number of likes, shares, comments, reactions, views, and/or website visits.

Kidney stone pain-related article selection

The results of the BuzzSumo search were screened to include only articles that met the predetermined standard of >50 engagements, then compiled and organized into appropriate categories. The top 10 articles with the most engagement were selected to evaluate the quality, understandability, actionability, and readability of these resources.

DISCERN instrument

The top 10 articles from BuzzSumo were assessed using the DISCERN instrument (Appendix 1). The DISCERN instrument is a validated tool that consists of 15 questions used to judge the quality of written health information and treatment choices [10]. The top 10 materials were independently read by four of the authors, and inter-rater agreement was determined using Cohen’s Kappa. The mean response from the authors was calculated to provide a concise view of the quality of each article.

Readability evaluation

Readability for the top 10 articles was also evaluated using an online readability tool called Readability Formulas [11]. An automatic readability checker on this site analyzes large bodies of text. The program examines the inputted text and calculates the number of sentences, words, syllables, and characters. These variables are then evaluated based on the criteria from seven contemporary readability formulas: the Flesch Reading Ease Formula, the Flesch-Kincaid Grade Level, the Fog Scale, the SMOG (Simple Measure of Gobbledygook) Index, the Coleman-Liau Index, the Automated Readability Index, and the Linsear Write Formula. Finally, a holistic reading grade level is generated for the sample text. The reading grade level for each article was recorded.

Patient Education Materials Assessment Tool (PEMAT)

PEMAT is an assessment tool that consists of 26 questions aimed at formulating a numeric value for the understandability and actionability of educational materials. This tool was used to assess the 10 materials in this study. Each question on the assessment tool is rated as “agree,” “disagree,” or “N/A,” where “disagree” receives a value of 0 and “agree” receives a value of 1. If a question is marked as N/A, it is eliminated from the total question count. The threshold for good versus poor understandability and actionability was set at 70%, based on previous publications.

## Results

Google Trends

**Figure 1:**
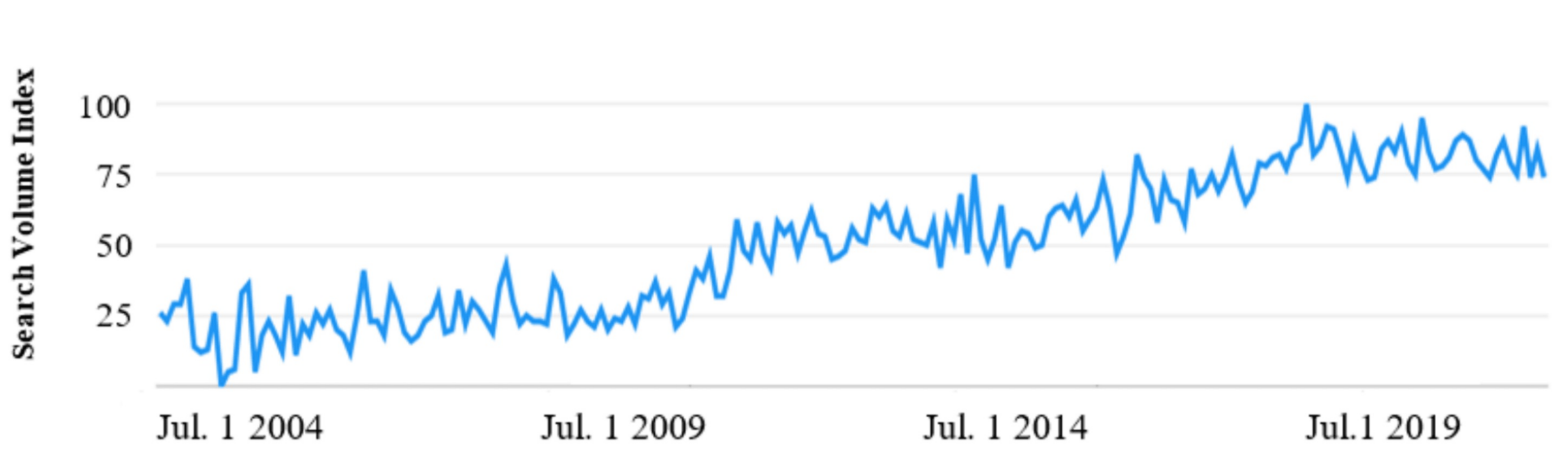
Google Trends displays the long-term search volume of the term “kidney stone pain.” Search volume index is a score relative to the highest peak on the graph; i.e., a score of 50 indicates search volume reached half of its peak.

The keyword “kidney stone pain” revealed an observable change over the last 16 years (Figure 1). Search volume has risen steadily since July 2004, when it was at its lowest. In August 2018, search volume reached its apex, and it has remained high to the present day. Kidney stone searches were found to focus on four main categories of questions: “passing stones,” “home remedies,” “stent pain,” and “back pain.”

BuzzSumo

Compiling and organizing the articles generated from the BuzzSumo searches (Table 1) revealed that the search phrase “passing stones” contained the most articles; therefore, it yielded the highest engagement across the web. Engagement is defined by the number of views that an article has received based on website visits or link clicks. The second most popular search category was “home remedies,” with 66.1k engagements, followed by “back pain” and “stent pain,” respectively.

**Table 1:** Most common engagements on kidney stone pain using the following four search categories: “passing stones,” “home remedies,” “stent pain,” and “back pain.”

	No. of articles	Total article engagement
Passing stones	28	439.1k
Home remedies	6	66.1k
Stent pain	1	143k
Back pain	7	2.9k

DISCERN instrument

The mean DISCERN score for the top 10 articles, calculated from the evaluations of all four reviewers, was 2.67/5. The highest-rated item was Q10 (Does it describe the benefits of each treatment?) with a score of 4.2/5. The lowest-rated item was Q11 (Does it describe the risks of each treatment?) with a score of 1.45/5. The inter-rater agreement score was 72%, meaning that the reviewers were substantially in agreement.

Readability evaluation

**Figure 2:**
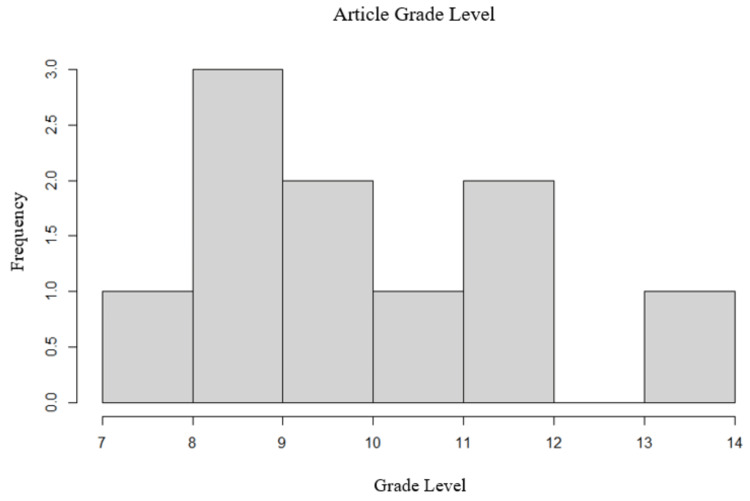
Mean reading grade level for the top 10 most popular articles.

An evaluation of readability revealed that the mean reading grade level was 10.4, while the median was 10 (Figure 2). Only one article fell below the eighth-grade reading level. Any article beyond the eighth-grade reading level was designated as “difficult to read.” One out of the 10 articles was written at the undergraduate level (grade 13). This article was recommended for a population that had completed studies at the collegiate level.

PEMAT (understandability and actionability) 

Three out of 10 articles reached the threshold of 70% to be considered “good” for understandability, with an average score of 60%. This means that the majority of the top 10 educational articles on kidney stone pain are not easily understood. Zero out of 10 articles reached the threshold of 70% to be considered “good” for actionability, with an average score of 31%.

## Discussion

The Google Trends results complement the existing literature that describes internet use as ubiquitous among patients [3,12]. We analyzed Google Trends data starting from 2004 to measure the overall trend of patients searching for online resources related to kidney stone pain, rather than focusing on specific time points within the data. Since 2011, searches for “kidney stone pain” on Google have rapidly increased, with the greatest search volume recorded in August 2018. The current search volume remains elevated and often approaches August 2018 levels. However, we expect searches related to medical information to increase as more people adopt online resources as their primary source of educational material. Patients younger than 65 years use the internet as a source of health-related information more frequently compared to those over 65 years - 93.8% vs. 66.3%, respectively [13,14]. This discrepancy will inevitably shrink over time as younger generations replace the current geriatric population as predominant internet users, bringing with them a greater reliance on the internet as a source of medical information. For this reason, online medical resources will become increasingly popular.

Since patients are expected to supplement their medical knowledge with online sources, the quality of these sources is an important factor. The DISCERN instrument is a validated rating tool used to evaluate the quality of health-related websites. The top 10 articles on “kidney stone pain” were assessed with a mean score of 2.67/5. According to the DISCERN grading system, this score represents “potentially important but not serious shortcomings.” This suboptimal evaluation is corroborated by other similar assessments of online medical information [12,15]. Herbert et al. reported that nearly three-fourths of online transcripts contained poor-quality content [12]; that is, they failed to mention sources of content, unbiased information, different treatment options, and associated risks and benefits of treatments. Comparably, an analysis of the top 10 articles determined using BuzzSumo reveals that they frequently describe the benefits associated with treatment but rarely or poorly describe the associated risks. SDM leads to better clinical outcomes in patients [16]; however, the top 10 articles also scored poorly on their support for SDM, with a mean score of 1.61/5. We analyzed BuzzSumo data starting from 2016, as this period immediately precedes the plateau observed in Google Trends data for kidney stone pain searches. While patients may be finding numerous sources online relating to kidney stone pain, the content’s understandability and readability are not reliable enough to support patients in SDM.

The purpose of this paper is to highlight the lack of quality educational materials available to patients and to call for a more standardized approach to designing and centralizing reliable online medical resources. While we are not providing specific examples of quality educational materials, we aim to emphasize key concepts to consider when evaluating the effectiveness of patient education resources. Such recommendations would be better suited for a separate paper, where implementation strategies can be thoroughly addressed. The quality of online articles needs to be improved; however, these improvements will be insufficient unless authors also consider the readability of their articles. The NIH and the CDC recommend that patient education material be written at or below the sixth- to eighth-grade level to cater to the average U.S. reading grade level [7]. The results of this study show that reality often does not conform to these standards. The readability software demonstrates that only 1 of the top 10 articles was at the recommended level, with the remainder at the high school or undergraduate level. Other studies evaluating online medical content in vascular surgery [17], respiratory medicine [18], and urologic medicine [12] also found poor readability scores. This discrepancy can be detrimental to patients, as they are often confused about the details of their disease and may be less willing to ask their physician for a thorough explanation [19]. In this regard, confusing and hard-to-read articles may negatively affect the kidney stone management plans of their readers.

In addition to improving the quality of online healthcare resources, understandability and actionability need to be considered when constructing these materials. The majority of articles failed to include structural components of writing, such as active voice, title breaks, and visual aids, which reduced their overall understandability score [20,21]. Compartmentalizing information improves understandability by encouraging readers to pause and digest what they have just read. Visual aids are also helpful because they enhance the reader's conceptualization of the material being presented [22].

Healthcare material should not only be educational but also provide patients with actionable items they can utilize to improve their overall condition. Only 1 article out of the 10 analyzed included actionable steps for patients to consider after the educational material was presented. Additionally, writing in the passive voice - as done in 8 out of 10 articles - as opposed to the active voice, disengages readers and makes it harder for them to remember crucial information [21-23]. The development of more comprehensive educational materials will ultimately improve patient outcomes, as patients will better understand their disease process.

To improve the quality of online patient educational resources, we should explore the role of artificial intelligence (AI) in designing personalized health content tailored to different patient populations, while prioritizing understandability, readability, and actionability. Healthcare systems should consider collaborating with tech companies to ensure the delivery of high-quality online medical information.

The internet offers a vast array of medical information. While one might expect that the most accurate resources would be those with the highest patient engagement, research suggests otherwise. Studies show that people are drawn to health information that is easy to understand, visually engaging, and accessible. For instance, during the COVID-19 pandemic, many turned to social media for updates. However, only a small group took extra steps to verify that information with reliable sources. Instead, most users found themselves gravitating toward content that was quick to digest - often in video form or visually appealing formats - rather than longer, more detailed articles that required higher health literacy. This preference often led people to engage more with sources that were easier to follow, even if they weren’t always the most scientifically accurate [24].

Another study from MDPI (Multidisciplinary Digital Publishing Institute) found that while people often act on health information they find online, they are usually drawn to sources with high production quality or peer recommendations, even if the information isn’t always scientifically accurate [25]. This tendency suggests that public health and medical organizations could benefit from tailoring their content to match the engaging formats commonly found in popular media. By doing so, they could more effectively compete with less reliable sources and have a greater impact on public health outcomes.

These findings emphasize the importance of future research focused on making accurate health information more appealing and accessible to a wider audience. To address this disparity, we aim to design a study that develops a standardized educational platform using the Delphi method to enhance patient understanding of medical conditions during hospital and clinic visits.

This study has several limitations. In line with the fast-paced nature of the internet, search results can quickly change in short periods. As articles are constantly posted and updated, our BuzzSumo results may be different from those of a search carried out at a different time. The readability software also has drawbacks that may hinder its precision. The program evaluates readability based on the number of syllables in a word and sentence length in the article, which may not be accurate representations of readability due to other variables, such as figures/images and page layout. Additionally, our internet searches were limited to English articles in the United States. We recognize that there is a large population of patients who do not speak English as their primary language, which is a topic of study we plan to cover in future work. Lastly, we acknowledge the abundance of online resources that include video content; however, we chose to limit our study to written materials, as there is currently no reliable tool available to assess the quality of video content.

## Conclusions

Urology patients increasingly rely on the internet for medical information, particularly regarding kidney stone pain. However, these articles generally exhibit medium to low content quality and are written at an inaccessible reading level, hindering patient comprehension. The urologic community should prioritize the creation of unbiased, reliable online resources. Additionally, efforts should focus on enhancing the clarity and practicality of educational materials to empower patients with a better understanding of their condition and to encourage autonomy.

## Appendices

Appendix 1

**Table 2:** DISCERN instrument

Question	Scoring
1. Are the aims clear?	Likert Scale (range = 1-5)
2. Does it achieve its aims?	Likert Scale (range = 1-5)
3. Is it relevant?	Likert Scale (range = 1-5)
4. Is it clear what sources of information were used to compile the publication (other than the author or producer)?	Likert Scale (range = 1-5)
5. Is it clear when the information used or reported in the publication was produced?	Likert Scale (range = 1-5)
6. Is it balanced and unbiased?	Likert Scale (range = 1-5)
7. Does it provide details of additional sources of support and information?	Likert Scale (range = 1-5)
8. Does it refer to areas of uncertainty?	Likert Scale (range = 1-5)
9. Does it describe how each treatment works?	Likert Scale (range = 1-5)
10. Does it describe the benefits of each treatment?	Likert Scale (range = 1-5)
11. Does it describe the risks of each treatment?	Likert Scale (range = 1-5)
12. Does it describe what would happen if no treatment is used?	Likert Scale (range = 1-5)
13. Does it describe how the treatment choices affect the overall quality of life?	Likert Scale (range = 1-5)
14. Is it clear that there may be more than one possible treatment choice?	Likert Scale (range = 1-5)
15. Does it provide support for shared decision-making?	Likert Scale (range = 1-5)
16. Based on the answers to all of the above questions, rate the overall quality of the publication as a source of information about treatment choices.	Average (mean) of the previous 15 questions
